# 

**Published:** 1897-10

**Authors:** 


### PERSONALS.

Dr. J. B. Hopper, recently in charge of the quarantine station at Garfield, N. J., has been transferred to the meat-inspection department of New York City. He is succeeded at Garfield by Dr. W. B. E. Miller, who was transferred from the New York department.

Dr. Charles W. Heitzman, of New Orleans, holds the post of chief of the meat-inspection service of Louisiana under the State Board of Health.

Dr. F. S. Schoenleber holds the position of dean of the McKillip Veterinary College. He is much interested in the work of the Association of Veterinary Faculties.

Dr. T. D. Hinebauch, of North Dakota, has been succeeded as instructor in the agricultural college and State Veterinarian by Dr. W. G. Langdon, of the same State.

Prof. Thomas Bowhill, of Edinburgh, Scotland, has been appointed chief government veterinary expert in Zululand, and will at once enter upon a thorough investigation of the present outbreak of rinderpest among the cattle of South Africa.

The enjoyment of the visit to Nashville was not wholly confined to the veterinary profession. Among the visitors was Mr. Frederick Krey, representing the firm of John Reynders & Co., of New York City, with an excellent display of veterinary instruments, which attracted a great deal of attention, and a very keen interest was espeically shown in the exhibition of a thermo-cautery and dental instrument destined to obviate many of the objections raised against instruments brought out to be used in similar work.

Dr. Robert C. Cliff, formerly of Hamilton, Canada, has located at Cavalier, North Dakota.

Dr. Leonard Pearson, of Philadelphia, in conjunction with his visit to Nashville, spent three days at Mammoth Cave, Kentucky.

Drs. J. F. Winchester, of Lawrence, and J. M. Parker, of Haverhill, Mass., included a visit to Mammoth Cave, Ky., while attending the thirty-fourth annual convention of the U. S. V. M. A.

Dr. E. B. Ackerman, of Brooklyn, has been selected as a veterinarian to the Humane Society of the City of Churches.

Drs. William Sheppard, of Sheepshead Bay, Long Island; Edward Loomes, and Thomas G. Sherwood, of New York City, have been selected as veterinarians to the thirteenth annual exhibition of the National Horse-show Association in Madison Square Garden, November 15 to 20, 1897.

Dr. E. L. Loblein, of New Brunswick, is one of the directors of the new Driving Park Association at New Brunswick, N. J.

Prof. Andrew Smith, of Toronto, Canada, was prevented from attending the convention at Nashville on account of the Industrial Exposition coming the same week. He is vice-president of the latter organization, and is general director of the live-stock exhibit.

Dr. Charles R. Wood, of Lowell, Mass., was one of the timers at the first performance in which the two-minute pace was lowered.

Veterinarian G. Howard Davison, of Millbrook, N. Y., was the winner of many prizes with his sheep shown at the New York State Fair.

Dr. F. S. Allen, of Philadelphia, was a visitor to Nashua, N. H., in the latter part of September.

Dr. W. R. Jobson, of Oil City, Pa., made a trip to Scotland in August last.

Dr. W. S. Kooker, of Philadelphia, met with a painful accident from falling, resulting in a severe scalp wound.

"Canadian Cattle and Their Inspection," by Dr. P. H. Bryce, is the title of an interesting article in the fifteenth annual report of the Provincial Board of Health of Ontario.

We are indebted to Dr. W. B. Niles for a copy of *Bulletin No.* 35 of the Iowa Agricultural College Experiment Station, in which we note several interesting articles on the feeding of lambs and calves, and an interesting review of the diseases of sheep in Iowa. An article on "Hog-cholera and Swine-plague," by Dr. W. B. Niles, adds much of interest from a review standpoint of the work done in this field. The book is profusely illustrated, and adds much of value to the contents and clearness of the text.

The many friends of Dr. H. D. Hanson will be glad to learn of his improved health and return to practise in New York City after a three-months' sojourn in the mountain regions of Sullivan County.

Dr. W. C. Carter, Professor of Physiology in the Veterinary Department of the University of Pennsylvania, has accepted the chair of physiology in the University of Texas.

The many warm friends of Dr. Isaiah Michener, of Carversville, Pa., will rejoice in knowing of his rapidly improving health from a recent light stroke of paralysis.

Dr. C. C. McLean, of Meadville, has made an enviable record for himself in directing and gaining for his city an excellent system of milk and dairy inspection.

The courtesy of Mr. Gray, representing Miller & Sibley, of the Prospect Hill Stock Farm, at Franklin, Pa., in extending so pleasant a trip to the members of the P. S. V. M. A. over this noted farm and among its wonderfully choice horses and cattle, will ever be a pleasant recollection to those who participated in this pleasure.

Dr. W. A. Chrisman, of Philadelphia, successfully passed the examination in April for inspector under the Bureau of Animal Industry.

Dr. John R. Mohler, of the Bureau of Animal Industry, has been transferred from Los Angeles, Cal., to Kansas City, Mo.

